# Highly Efficient Generation of Transgenic Sheep by Lentivirus Accompanying the Alteration of Methylation Status

**DOI:** 10.1371/journal.pone.0054614

**Published:** 2013-01-29

**Authors:** Chenxi Liu, Liqin Wang, Wenrong Li, Xuemei Zhang, Yongzhi Tian, Ning Zhang, Sangang He, Tong Chen, Juncheng Huang, Mingjun Liu

**Affiliations:** 1 Xinjiang Laboratory of Animal Biotechnology, Urumqi, Xinjiang, China; 2 Key Laboratory of Genetics, Breeding and Reproduction of Grass Feeding Livestock, Ministry of Agriculture, Urumqi, Xinjiang, China; 3 Animal Biotechnology Research Center, Xinjiang Academy of Animal Science, Urumqi, Xinjiang, China; 4 College of Life Science and Technology, Xinjiang University, Urumqi, Xinjiang, China; The Walter and Eliza Hall of Medical Research, Australia

## Abstract

**Background:**

Low efficiency of gene transfer and silence of transgene expression are the critical factors hampering the development of transgenic livestock. Recently, transfer of recombinant lentivirus has been demonstrated to be an efficient transgene delivery method in various animals. However, the lentiviral transgenesis and the methylation status of transgene in sheep have not been well addressed.

**Methodology/Principle Findings:**

EGFP transgenic sheep were generated by injecting recombinant lentivirus into zygotes. Of the 13 lambs born, 8 carried the EGFP transgene, and its chromosomal integration was identified in all tested tissues. Western blotting showed that GFP was expressed in all transgenic founders and their various tissues. Analysis of CpG methylation status of CMV promoter by bisulfate sequencing unraveled remarkable variation of methylation levels in transgenic sheep. The average methylation levels ranged from 37.6% to 79.1% in the transgenic individuals and 34.7% to 83% in the tested tissues. Correlative analysis of methylation status with GFP expression revealed that the GFP expression level was inversely correlated with methylation density. The similar phenomenon was also observed in tested tissues. Transgene integration determined by Southern blotting presented multiple integrants ranging from 2 to 6 copies in the genome of transgenic sheep.

**Conclusions/Significance:**

Injection of lentiviral transgene into zygotes could be a promising efficient gene delivery system to generate transgenic sheep and achieved widespread transgene expression. The promoter of integrants transferred by lentiviral vector was subjected to dramatic alteration of methylation status and the transgene expression level was inversely correlative with promoter methylation density. Our work illustrated for the first time that generation of transgenic sheep by injecting recombinant lentivirus into zygote could be an efficient tool to improve sheep performance by genetic modification.

## Introduction

The generation of transgenic livestock holds considerable promise for the development of biomedical and agricultural systems [1], [2]. The first transgenic livestock was produced via microinjection of foreign DNA into pronuclei of zygote in 1985 [3]. In 1986, cloning sheep was generated by nuclear transfer using embryonic stem cells as donors [4], and then cloning sheep Dolly was born in 1997 by somatic cell cloning (SCC) [5]. Concomitant with the success of SCC, the first cloning transgenic sheep was produced by nuclear transfer with stably transgenic somatic cells. In spite of the success in generation of transgenic livestock by pronuclear microinjection or SCC, concurrent techniques shows several significant shortcomings, such as low efficiency, high cost, random integration, and frequent incidence of mosaicism. Efficient generation of transgenic livestock with low cost remains to be developed in transgenic animal field. Recent development of lentiviral vector for gene transfer shows the great potentials to overcome limitations mentioned above [6], [7], and accordingly is becoming a new efficient tool to produce transgenic livestock.

To date, various transgenic species including mice, fish, chicken, pig, non-human primate, cattle and sheep have been generated by lentiviral transgenesis [8], [9]. Compared to traditional pronuclear DNA microinjection or somatic cell cloning (SCC), lentiviral transgenesis results in a four to eight fold higher generation rate of transgenic animals per embryo treated [10], and more than 90% transgenic founders can be observed transgene expression [11], [12]. Furthermore, the transgene delivered by lentiviral vector also stably expressed in their offsprings with considerably low methylation level in transgene promoter under certain circumstances. This differed from retrovirus-induced globally *de novo* methylation, which resulted in widespread silence of transgene expression [13].

Transgenic swine was the first livestock produced by injecting lentivirus into zygote with generation rates of 19–33% [14], which was significantly higher than 1% such rate obtained by conventional pronuclear microinjection [3]. However, the same investigators who successfully introduced lentiviral transgene into swine failed to produce transgenic cattle by the same procedure although the transgenic embryos were gained [15]. In 2004, the first transgenic cattle was produced by lentivirus infection of oocyte instead of microjection with the generation rate of 8.3% per oocyte treated [15]. In 2012, the transgenic cattle generated by injection of lentiviral vector into zygotes was reported with the generation rate of 7.5% per embryo transferred [16]. These studies indicated that the infection and integration capability of recombinant lentivirus were quite disparate within different livestock species.

Previous studies on lentiviral transgenesis demonstrated that the transgene expression was associated with transgene epigenetic modification, integrant numbers and locus [17], [18]. So far, the overall regulatory mechanism of lentiviral transgene expression has been poorly understood. DNA hypermethylation was considered as a critical factor resulting in silence of transgene expression. Concurrent report also showed that about one-third of integrated lentiviral transgenes in pigs were subjected to methylation and exhibited lower expression [19].

As for transgenic sheep, since the first one was produced in 1985 by Hammer with pronuclear microinjection [3], some new approaches have been used for production of transgenic sheep, for instance, somatic cell cloning (SCC) [5] and sperm-mediated gene transfer (SMGT) [20]. In the mass, the transgenic efficiency remains extremely low. Ritchie *et al* succeeded in producing transgenic sheep by transferring blastocysts derived from oocyte injection of lentivirus with 20% transgenic efficiency [21]. However there are few comprehensive studies on the diversity of transgene integration, expression and alteration of methylation in transgenic sheep. Especially, transgene efficiency, expression pattern and epigenetic state of transgenic sheep produced by lentiviral injection have not been well understood. Hereby, we demonstrated for the first time that the transgenesis by injection of EGFP-lentivirus into perivitelline space of sheep zygote is a high efficient tool for generation of transgenic sheep and transgene can be expressed in almost all transgenic founders and their various tissues. Furthermore, methylation status of transgene and its effect on transgene expression, as well as relationship between integrant numbers and its expression were firstly investigated in lentivirus-mediated transgenic sheep.

## Materials and Methods

### Animals

All animals used for this study are Xinjiang Merino Fine Wool Sheep raised in the farm of Sheep Breeding and Reproduction Center. All studies carried out in sheep were approved by the Committee of Animal Research Security and Ethics (CARSE), Xinjiang Academy of Animal Science.

### Construction of Plasmids and Preparation of Lentiviral Particles

EGFP gene was digested from pEGFP-N1 plasmid (Clontech) with *BamH I* and *Hind III* (TAKARA) and cloned into lentiviral vector (pLEX-MCS, Openbiosystem), named as pLEX-EGFP. The vector carries self-inactivating long terminal repeat (SIN LTR), internal ribosome entry site (IRES), mammalian selectable marker (puromycin) and woodchuck hepatitis virus posttranscriptional regulatory element (WPRE). EGFP gene is located at downstream of the CMV promoter.

**Table 1:** Southern blot analysis of transgene copy numbers determined by standard curve with a double-digested genomic DNA sample.

Transgenic Sheep	#4	#5	#6	#7	#8	#9	#12	#14
Intensity	931	1949	1362	952	982	1013	2222	1442
Copy Numbers	1.88	4.99	3.2	1.94	2.03	2.13	5.83	3.44

To generate pLEX-EGFP lentiviral particles, HEK293T cells were seeded in a 100-mm dish at a density of 60,000 cells/cm^2^ and co-transfected with pLEX-EGFP (12 µg) along with packaging plasmids (3.5 µg pMD2.G and 6 µg pPAX2) using Lipofectamine 2000 (Invitrogen) at a DNA/Lipofectamine ratio of 1 to 3. After 48 h transfection, the supernatant containing lentivirus particles was filtered through 0.45-µm syringe filter and concentrated by ultracentrifugation (Beckman) at 50,000 g for 2 hour at 4°C. Precipitation was resuspended in phosphate buffered saline, aliquoted and stored at −80°C. Lentivirus titre was determined by infecting HEK293T cells with serial dilutions of concentrated lentivirus, and thereafter quantitated by counting the GFP fluorescent cells with flow cytometry (Becton, Dickinson and Company) post of 48 h infection as previous described [22]. The titre of pLEX-EGFP was approximately 3×10^9^ infectious units (IU) per ml in average.

### Lentivirus Injection and Embryo Transfer

Transgenic sheep were generated via injection of lentivirus into perivitelline space of the zygote. In brief, embryos were obtained from Xinjiang Merino Sheep which were approximately 2 years old and weighed at least 50 kg. Superovulation was carried out within sheep breeding season from September to November and started on 3 days before oestrus induced by intramuscular injection of follicle stimulating hormone (FSH, Sigma-Aldrich). FSH was injected once per 12 hours lasting for 3 days. Briefly, twice injection of 40 IU FSH was performed on the first day, 30 IU on the second day and 20 IU on the last day. After 12 hour of oestrus, the donors were mated with rams and repeated mating another 12 hours later. At 60 hours, zygotes from mated donors were collected by flushing the umbrella of oviducts with warm phosphate buffered saline containing 2% FBS. Then they were removed from the PBS and cultured in SOF medium with 3 mg/mL BSA at 38°C in 5% CO_2_.

For lentivirus injection, around 50–100 pl of concentrated lentivirus with 3×10^9^ IU/ml titer were injected into perivitelline space of zygotes using a micromanipulator (ECLipse TE2000-U, Nikon). For embryo transfer, recipients were synchronized by the same treatment as donor ewes. Embryos injected at one or two cell stage were transferred to recipient ewes with mid-line laparotomy under general anaesthesia. During surgery, the reproductive tract was exposed and embryos were transferred into the oviduct of recipients using a displacement micropipette. To assess the expression of GFP in vitro, part of injected zygotes were cultured to blastula in SOF medium supplemented with 3 mg/ml BSA at 38°C in 5% CO_2_ and observed under UV-microscope.

### PCR Detection

Transgene integration was detected by PCR screening. Genomic DNA was obtained from tail tips using the DNeasy@ Blood and Tissue Kit (QIAGEN) according to the instruction manual. PCR analysis was carried out with 500 ng genomic DNA as template and PCR Master mix (Promega). Primers used to amplify the 638 bp transgene fragment spaning CMV prompter and EGFP gene were: forward 5′-CACCAAAATCAACGGGACTT-3′ and reverse 5′-GATGTTGCC GTCCTCCTTGAAGT-3′. The PCR conditions were 94°C denaturation for 5 min followed by 40 cycles of 94°C for 30 sec, 60°C for 45 sec, and 72°C for 55 sec and a final extension at 72°C for 7 min.

### Southern Blotting

Integration numbers of transgene were determined by Southern blotting analysis. Genomic DNA from tail tips was extracted by means of standard phenol-chloroform extraction and digested with *Eco*RI (TAKARA) or double-digested with *SfiI* and *HpaI* (TAKARA). After precipitation with alcohol, 10 µg digested DNA was separated on 0.7% agarose gel with 25 volt electrophoresis overnight. Blotting was carried on by vacuum transfer to nylon membrane (Amershan) in 10×SSC for 90 min. The 430 bp fragment of the CMV promoter was amplified as probe from pLEX-EGFP plasmid using primers: forward 5′-CGAGGGCGATGCCACCTAC-3′ and reverse 5′-CTCCAGCAGGACCATGTGATC-3′. The probe was prepared by ^32^P-dCTP labeling with random primer extension kit (Promega) and hybridized with blotting membrane by incubating overnight at 65°C in hybridization oven (Hoefer Scientific Instrument). The concentration of probe used for hybridization was 25 ng/µL. Membranes were washed three times at 65°C in 0.5×SSC buffer containing 1% SDS after hybridization and exposed against film in dark cassette at −80°C for 24 hours. Then the film was developed as general protocol.

To verify the integrant numbers observed in one-cut genomic DNA, the southern blot with double-digested genomic DNA was performed along with the standard curve which was generated by serial dilution of double-digested transgenic plasmid in parallel. For short, the plasmid was serially diluted from 120 pg (5 copies) to 24 pg (one copy). Each concentration of standard plasmid was converted into copy numbers per volume using the following equation: *N = 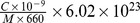,* where *N* stands for copy number (copies/µL), C for concentration (ng/µL) and M for base pairs of the plasmid. Further, the integrants identified by counting the bands in single-digested genomic DNA southern blot was matched to the copy numbers determined in double-cut genomic DNA southern blot by quantification with standard curve.

### Fluorescence Imaging

Photomicrographs of embryo were taken under fluorescent microscope (ECLipse TE2000-U, Nikon) using Nis-Elements software. For transgenic sheep, GFP images were performed with a Wd-9403e UV portable device (61 Biological Instrument, Peking) fitted with UV filter and captured using a 5D-Mark 2 digital camera (Canon, 50 mm lens).

### Western Blotting

Total proteins were extracted from tail tips or other tissues. Frozen samples were ground to powder by pestle and mortar grinding and solubilized in a solution of 62.5 mM Tris pH6.8, 10% glycerol, 2.5% sodium dodecyl sulfate (SDS), and Halt^TM^Protease Inhibitor Cocktail (Thermo Scientific). Quantification of total protein was carried out by Bicinchoninic acid assay with BSA (Sigma-Aldrich). The proteins (100 µg) were subjected to 12% SDS-polyacrylamide gel electrophoresis. Separated proteins were transferred to nitrocellulose (NC) membrane (Bio-Rad) and immune-blotted with anti-GFP or anti-β-actin antibodies (Abcam). Immuno-reactive proteins were visualized using the Odyssey Infrared Imaging System and relatively quantified by densitometric analysis (Li-Cor, Lincoln, NE), as described by the manufacturer.

### Bisulfite Sequencing

The genomic DNA was extracted from tail tips or other tissues by DNeasy@ Blood&Tissue Kit (QIAGEN) according to the instruction manual. Bisulfite modification was performed with 0.6 µg of DNA for each sample using the EpiTect@Bisulfite Kit (QIAGEN) according to the instruction manual. PCR primers used to amplify the CMV promoter were designed by MethPrimer software online (http://www.urogene.org/methprimer/), which was also used to predict CpG site and CpG islands. The following PCR primers were used to amplify a 487-bp fragment containing one CpG islands with 30 CpGs: forward 5′-GGGTTATTAGTTTATAG TTTATATATGG-3′ and reverse 5′-GATTCACTAAACCAACTCTACTTA-3′. The PCR of bisulfite-modified DNA was performed using PCR Master MIX (Promega). Amplicons were gel purified and cloned into pGEM-T vector (Promega), followed by sequencing at least 7 clones of each sample. For each DNA sample, the number of cytosine residues that remained as “C” was counted, and converted to a percentage of the total 30 CpGs presented in 487 bp region of the CMV promoter.

### Statistical Analysis

Statistical analysis were performed with the SPSS 13.0 software. Values were shown as Mean±SD and subjected to correlation analysis of Pearson. *P* value less than 0.05 was considered as statistically significant.

## Results

### Generation of EGFP Transgenic Sheep

**Figure 1:**
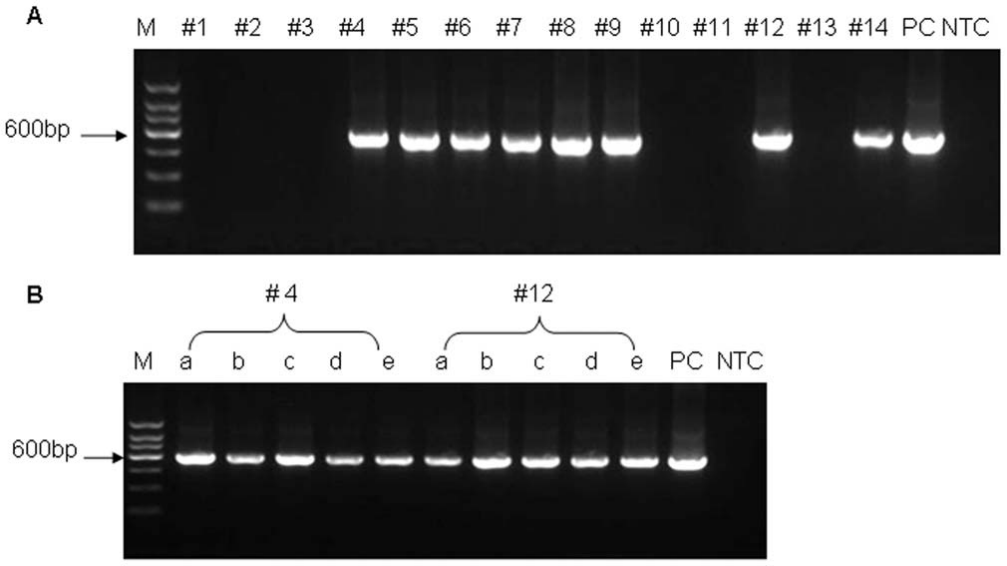
Analysis of EGFP-lentivirus transgene integration in transgenic sheep. (A) Amplification of EGFP transgene from genomic DNA extracted from tail tips of newborn lambs. #1–14: transgenic newborn lambs. (B) Amplification of EGFP transgene from tissues of #4 and #12 anatomized lambs. a-e: heart, liver, spleen, lung and kidney, respectively. Amplicons are 604 bp fragments spanning CMV promoter and EGFP sequences. M, DNA marker; PC, pLEX-EGFP vector as positive control; NTC, non-transgenic sheep DNA control.

**Figure 2:**
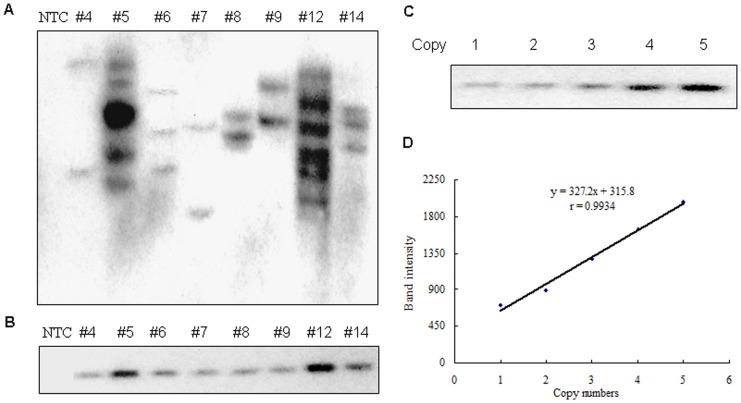
Southern blotting analysis of transgene integrants in genomic DNA of transgenic sheep. (A) Genomic DNA extracted from tail tips of transgenic sheep was digested with *EcoRI* and hybridized with ^32^P labeled probe amplified from CMV promoter. (B) Genomic DNA extracted from tail tips of transgenic sheep was double-digested with *Sfi*I/*Hpa*I and hybridized with ^32^P labeled probe. NTC, non-transgenic sheep control; # 4–14, transgenic lambs identified by PCR corresponding to Fig. 1A. (C) pLEX-EGFP plasmid was double-digested with *Sfi*I/*Hpa*I and diluted in serial concentrations matched to corresponding copies. Diluted plasmids with copies from 1 to 5 were hybridized with probe double-digested genomic DNA of transgenic lamb in parallel. (D) Standard curve of copy numbers in panel C was generated with diluted plasmid based on the quantification of the blots by densitometric measurement as described in the Materials and Method.

Total of 46 zygotes were collected from FSH stimulated donors after artificial insemination. One or two cell stage embryos were injected with lentivirus (3.7×10^9 ^IU/mL) into perivitelline space. The injected embryos were then transferred to 22 hormonally synchronized recipients. In order to increase the productivity, all recipients were transferred with two embryos and resulted in the birth of 13 lambs from 9 pregnant ewes. Of the 13 newborn lambs, eight transgenic sheep were identified by PCR (Fig. 1A) and southern blotting (Fig. 2A and 2B). The rate of transgenic sheep to total of new born lambs and to embryos were 61.5% (8/13) and 17.4 (8/46) respectively. Except two lambs (#4 and #12) died after birth, the other 6 lambs survive normally. There was obvious variation of congenital malformation in dorsal keel of #4 lamb with death at birth. The other died lamb #12 displayed the anorexia and diarrhea before death, no other developmental abnormality was observed. Transgenic sheep mortality is 25% (2/8), which is the same as that of normal lamb of 25% (9/36). For the two died transgenic lambs, the genomic DNAs extracted from heart, liver, spleen, lung and kidney were subjected to PCR screening. The integration of transgene was observed in all tested tissues (Fig. 1B). The results inferred that the integration of lentiviral transgenesis may exist in all the tissues.

### Analysis of the Transgene Integration

In order to analyze transgene integration and copy numbers, southern blot assay was carried out with genomic DNA digested with *EcoRI* or double-digested with *SfiI* and *HpaI*. In *EcoR*I digested genomic DNA samples, the number of integrants were visualized ranging from 2 to 6 copies and for most individuals with 2 to 3 copies (Fig. 2A). To exactly quantify the copy number of each transgenic sheep, we performed the southern blot with double-digested genomic DNA and quantified the copy number by standard curve. The standard curve was generated with pLEX-EGFP plasmid by concentration gradient southern blot, which was performed in parallel with double-digested genomic DNA derived from transgenic lambs (Fig. 2B). The copy number of each double-digested plasmid with serial dilution was linearly matched to the plasmid concentration (Fig. 2C and 2D). The copy number for each blot of transgenic lamb was calculated based on standard curve (Table 1).

The highest copy number was identified in #12 lamb with 6 copies, followed by #5 lamb with 5 copies. The copy numbers of other transgenic sheep were around 2 to 3. Copy number derived from these two approaches was consistent (Fig. 2A).

### Analysis of EGFP Expression in Transgenic Lambs

**Figure 3:**
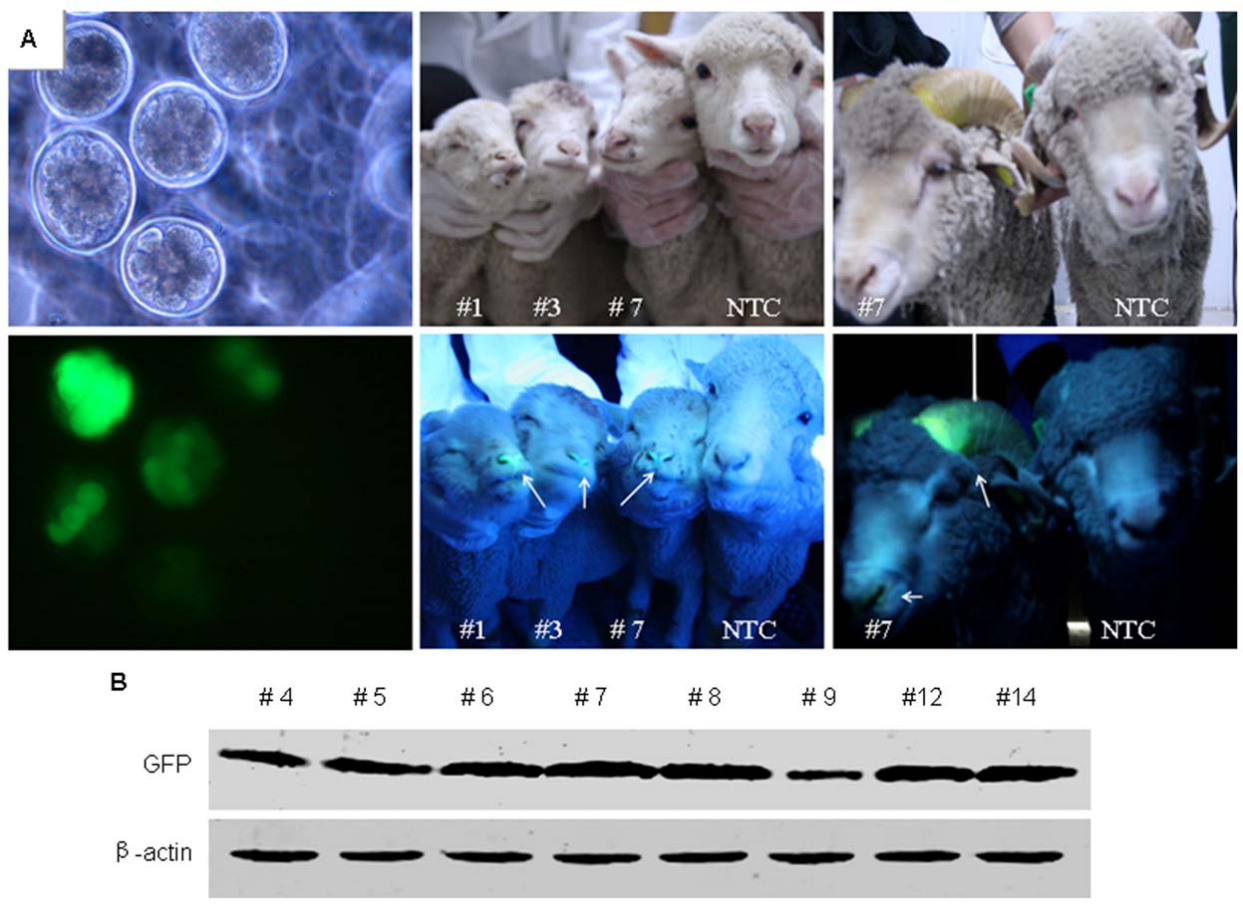
Analysis of the expression of GFP in transgenic lambs. (A) Embryos injected with lentivirus were cultured and developed to blastula and visualized by white and UV light (left panels) under microscope with magnification of 200×. Visualization of GFP expression in transgenic lambs (#1,#3,#7) and non-transgenic lamb control (NTC) were pictured under white light and UV light (middle panels). Visualization of GFP expression of horn in 1.5 year old transgenic lamb #7 and non-transgenic lamb pictured under white light and UV light (right panels). Arrows indicated the green fluorescence in transgenic sheep; (B) Proteins extracted from tail tips of eight transgenic lambs were subjected to immunoblotting with GFP antibody as described in Materials and Methods. β-actin levels were determined with an anti-β-actin antibody and used as loading control.

**Figure 4:**
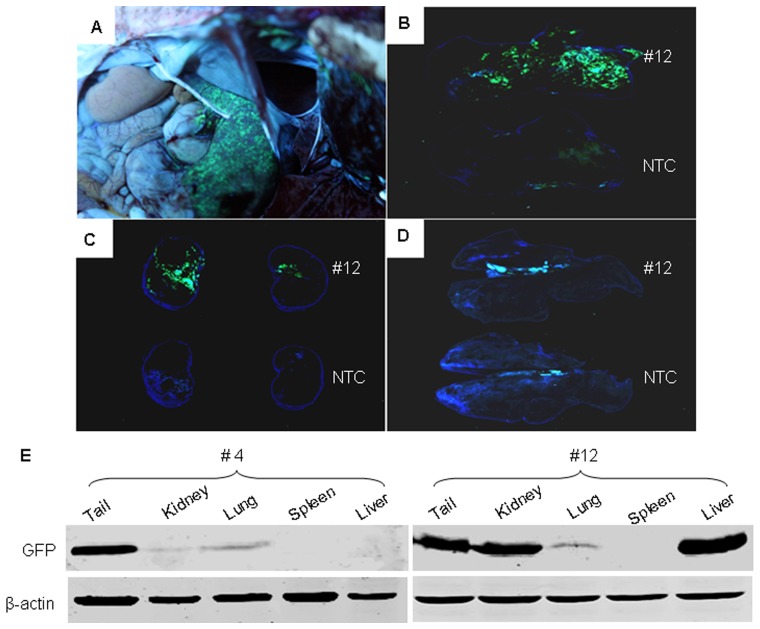
Expression of GFP in tissues of transgenic lambs observed by fluorescence imaging and assayed by western blotting. (A) Fluorescence imaging of the whole inner organs of transgenic sheep under white light. (B-D) Fluorescence imaging of liver, kidney and lung of transgenic or NTC sheep. The upper are organs of the transgenic sheep and the lower are organs of the NTC sheep. (E) Expression of GFP in tissues assayed by western bloting. Proteins extracted from tissues of tail tip, kidney, lung, spleen and liver of #4 and #12 lambs were subjected to immunoblotting with GFP antibody as described in Materials and Methods. β-actin levels were determined with an anti-β-actin antibody as loading control.

The expression of EGFP transgene was analyzed by direct fluorescence observation and Western blotting. At first, we observed embryos injected with EGFP lentivirus in blastula stage under fluorescent microscope (Fig. 3A, left panels). Approximately 80% embryos subjected to injection of lentiviral transgene were presented green fluorescence. Further, we observed green fluorescence in hoof, lip and horn of newborn transgenic lambs (Fig. 3A, middle panels) and continuously to maturity (Fig. 3A, right panels), which suggested that the GFP could be expressed persistently in transgenic sheep. Additionally, we anatomized the died lamb (#4 and #12) to investigate the distribution of GFP expression in inner organs (Fig. 4A). Notably, the most intense GFP fluorescence was observed in liver (Fig. 4B) and then in kidney (Fig. 4C), weak GFP fluorescence was observed in lung of #12 lamb (Fig. 4D). To further analyze the GFP expression, we extracted the proteins from tail tips of all transgenic sheep and inner organs from two died lambs to perform Western blotting. Expression of GFP was detected in tail tips of eight transgenic lambs (Fig. 3B), which indicated that GFP transgene expressed in all transgenic founders. The relative quantification of western blot showed that the levels of GFP expression of #7 and #8 were much higher than that of other founders. Consistent with green fluorescent intensity in inner organs of #12 lamb, the level of GFP protein measured by western blotting was highest in liver and lowest in lung, no GFP was detected in spleen (Fig. 4E, right panel). The expression of GFP in lamb #4 indicated that the expression of GFP was highest in tail and lower in lung and kidney, and no expression was detected in spleen and liver (Fig. 4E, left panel). These data indicated the disparity of transgene expression in different individuals and tissues.

### Status of Promoter Methylation and Influence on Transgene Expression

**Figure 5:**
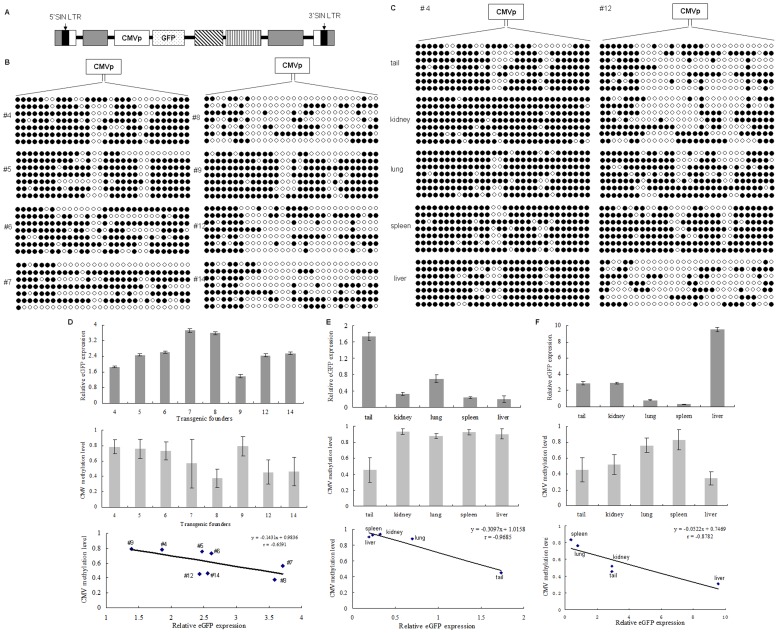
Correlation of CMV promoter methylation status with GFP expression level of transgenic sheep. (A) Schematic of pLEX-EGFP vector. (B) Status of the CMV promoter methylation in 8 transgenic sheep. The 487bp sequences of CMV promoter containing one CpG islands were targeted for methylation analysis. Genomic DNAs extracted from 8 transgenic lambs (#4–12) were treated with bisulfite and sequenced at least 7 clones for each sample. (C) Status of the CMV promoter methylation in tested tissues of two anatomized lambs (#4 and #12). Genomic DNAs extracted from tail tips, liver, lung, kidney and spleen were treated with bisulfite and sequenced at least 7 clones for each sample. The black cycles represented the methylated CpG and the white cycles represented the non-methylated CpG. **(**D) Correlation of GFP expression with methylation level of lentiviral CMV promoter of transgenic lambs. Densitometric quantification of the relative GFP expression was assayed by Western blotting (Fig. 3B) in tail tips of transgenic lambs #4–14 (up panel). Methylation levels were measured by the average ratios of methylated CpGs to total CpGs of the target CMV promoter sequence (middle panel). Correlation of the methylation levels of CMV promoter with GFP expression of 8 transgenic sheep was analyzed (low panel). (E and F) Correlation of GFP expression with methylation levels of CMV promoter in tested tissues of anatomized lambs (#4 and #12). Densitometric quantification of the relative GFP expression was assayed by Western blotting (Fig. 4B) in tissues of #4 (E, up panel) and #12 (F, up panel) lamb. Methylation levels of CMV promoter in tested tissues of #4 (E, middle panel) and #12 (F, middle panel ) lambs were based on Fig. 5C. The average rate of methylated CpGs in the 487 bp region of CMV promoter was defined as the indicator of methylation status. Correlation of methylation levels of CMV promoter with GFP expression levels was analyzed in tested tissues of #4 (E, low panel) and #12 (F, low panel) lambs.

Previous studies documented that transgene could be methylated in transgenic animals and resulted in repression of expression [23], [24]. To investigate the methylation status and its influence on transgene expression in lentiviral-mediated transgenic sheep, we examined the methylation density of 487-bp region of the CMV promoter containing one CpG island with 30 CpGs in individuals and tissues. Firstly, CMV promoter methylation status in all transgenic founders was measured (Fig. 5B). The average methylation levels ranged from 37.6% to 79.1% in transgenic individuals (Fig. 5D, middle panel). Then the promoter methylation status in different tissues was measured (Fig. 5C) and the methylated CpG rate ranged from 34.7% to 93.3% (Fig. 5E and 5F, middle panels). Analysis of the correlation of methylation level with GFP expression in individuals (Fig. 5D, low panel) and tissues (Fig. 5E and 5F, low panels) revealed that the expression of GFP expression was inversely correlated with methylation status (r = - 0.6591 for individules, *p*<0.05; r =  −0.9685 for #4 tissues, *p*<0.05; r =  −0.8782 for #12 tissues, *p*<0.05). The lowest GFP expression (Fig. 5D, up panel) was observed in the transgenic sheep with the highest methylation level (79.1%, transgenic sheep #9). On the contrary, the lowest promoter methylation level was corresponding to the highest GFP expression level (transgenic sheep #8). In tissues, the highest methylation level was found in spleen of #4 lamb with 93.3% (Fig. 5E, middle panel), at which little GFP expression was detected (Fig. 5E, up panel), whereas the highest expression of GFP was found in liver of #12 lamb (Fig. 5F, up panel), in concomitant with the lowest methylation density (34.7%) (Fig. 5F, middle panel).

## Discussion

Concurrent studies documented that lentiviral vectors had been successfully used to generate transgenic mice, rat, pig, cattle, chicken and nonhuman primate [8], [14], [25], [26], [27], [28]. Different transgenic species generated by lentiviral vectors exhibited variability in gene transfer efficiency, transgene expression and epigenetic status. In this study, we generated 8 transgenic sheep by injection of lentiviral vector containing EGFP reporter into perivitelline space of ovine embryos with 17.4% transgenic efficiency, which was substantially higher than that of cattle produced using same method with rate of 7.5% (3/40) [16]. Previous reports on transgenic mice indicated that lentiviral injection should be performed at one-cell stage of zygotes [22], [29]. As the variegation of response on the effect of superovulation treatment among donors, it is difficult to maintain the collected sheep embryos in the same stage. In our studies, approximate 60% of zygotes gained were on one-cell stage, and the other stayed on two-cell stage. Based on our in vitro study by injection of GFP into IVF embryos at different stages, there is no significant difference of transgenic efficacy between one-cell and two-cell stage (76.9% versus 75.4%, data not shown). For the two lambs died postnatal, one (#4) was found with over-bend dorsal keel. The other lamb (#12) displayed the anorexia and diarrhea, which were the major causal that the non-transgenic sheep died from. The ratio of mortality was 25% in transgenic lambs, whereas the mortality of wild type investigated in the same reproductive term was 25% (9/36). There is no difference in mortality between transgenic sheep and non-transgenic sheep, which indicated lentiviral transgenesis has no obvious disturbance on development of transgenic sheep.

Multiple copies of integration are substantially observed in transgenic animals produced by lentiviral transgenesis [27], [30]. Based on our analysis of lentiviral integration, we found that lentiviral transgene was occurred in various tissues of transgenic sheep. Moreover, the southern blotting illustrated that most of the transgenic lambs possessed more than one copy of integrant. The average integrant numbers were 3.1 (25/8), ranging from 2 to 7. Depending on literature investigation, the average integrant numbers in transgenic pigs generated by injection of recombinant lentivirus were 4.6, ranged from 1 to 20 copies [14]. The variability of integrant numbers was presumably associated with animal species and lentiviral titer injected.

Previous investigators had addressed unprecedented high rate of transgene expression [11], [12]. To investigate the transgene expression, observation on whole lambs showed green fluorescence in hoof, lip and horn from birth continuously to maturity. Tissues from freshly dead transgenic lambs also presented green fluorescence in liver, kidney and lung. This was consistent with the results reported in pigs and cattles [15], [31]. To further verify the expression of transgene, the proteins extracted from different tissues were subjected to western blotting analysis. The GFP protein expression varied among individuals and tissues of transgenic sheep, which was in consistence with the fluorescent intensity observed in vivo fluorescence imaging. In general, the overall expression of transgenic sheep derived from lentiviral transgenesis indicated the wide range of transgene expression in different individuals. However, dramatic disparity of transgene expression was identified in different tissues. Our results were similar to the report in transgenic pigs and birds [19], [32]. Variegation of transgene expression might be explained by differences in the basic biology or lentiviral vector activity. On the other hand,these results were obtained from F0 founders. Based on previous report in pig [20], one-third of lentivirus-mediated transgenic pigs of F1 generation exhibited low expression levels and hypermethylation. Further studies are worthwhile to carry out to investigate the transgene expression in F1 generation of transgenic sheep in the future.

Previous reports showed that DNA methylation has been verified as a critical factor in regulating activity of transgenic vector [23], [24], [33]. Meanwhile, analysis of methylation status of transgenic pigs found that the high degree of methylation in the promoter and coding region of lentiviral transgene was accompanied by low levels of transgene expression [20]. To explore the regulation mechanism of lentiviral transgene, we measured CMV promoter methylation levels and analysed the association of promoter methylation with transgene expression in all transgenic founders and part of tissues. Our results showed that the methylation levels ranged from 37.6% to 79.1% in transgenic individuals and 34.7% to 83% in tested tissues. The association of methylation level with GFP expression suggested that GFP expression was inversely correlated with methylation status, both in individuals and in tissues. This result was similar to the outcomes reported in transgenic mice [34] and pigs of somatic cell cloning [33]. The number of lentiviral transgene integrant was reported as an important factor involved in transgene expression besides the methylation [35]. Studies on lentiviral transgenic pigs found that the increase of transgene expression was almost linearly increasing with lentiviral integrant numbers [14]. In this study, there is no any inclination between integrant numbers and GFP expression levels (r  = 0.128, p>0.05, datas not shown). We postulated that the lentiviral transgene expression was presumably influenced by integration loci and its context rather than integrant numbers. Further studies have been carried out to survey the integrant loci and study the association with transgene expression. Our results also inferred that the level of promoter methylation played much more important role in controlling transgene expression than that of integrant number in lentivirus-mediated transgenic sheep.

Since the first publication on generation of transgenic sheep by injection of lentivirus into oocytes in 2009 [21], no further studies have been reported so far. Hereby, we are the first time to comprehensively investigate the issues of transgenic integrant, expression and methylation in lentivirus-mediated transgenic sheep. Taken together, we demonstrated that lentiviral transgenesis by injection of recombinant lentivirus into perivitelline space of sheep zygote could achieve high transgenic efficiency and high rate of transgene expression. Furthernore, the lentiviral transgene was subjected to alteration of methylation status and the transgene expression was inversely correlative with promoter methylation, whereas has no association with integrant numbers in lentivirus-mediatied transgenic sheep.
